# Supplementary material for: Environmental Risk Factors for Childhood Acute Lymphoblastic Leukemia: An Umbrella Review
Source: Cancers (Basel). 2022 Jan 13;14(2):382. doi: 10.3390/cancers14020382 (PMC8773598; doi:10.3390/cancers14020382)
Supplement: Supplementary file 1 [file cancers-14-00382-s001.zip › Sup - TableS1.pdf]

Table S1: Quality assessment and risk of bias in systematic reviews using A Measurement Tool to Assess Systematic Reviews (AMSTAR 2).

Author, Year	PECO[1]	A priori Methods[2]	Study Design Selection[3]	Search Strategy[4]	Study Selection[5]	Data Extraction[6]	Excluded Studies[7]	Included Studies[8]	RoB Assessment[9]	Funding Sources[10]	Statistical Methods[11]	Impact of RoB[12]	RoB Results[13]	Heterogeneity[14]	Publication Bias[15]	Conflict of Interest [16]	Overall Rating[17]
Baker and Hoel, 2007	Y	N	Y	PY	N/A	N/A	N	PY	Y	Y	Y	Y	Y	Y	Y	Y	Low
Veisani and Delpisheh 2018	Y	N	N	N	Y	Y	Y	Y	Y	N	Y	Y	Y	Y	Y	N	Low
Lu et al., 2020	Y	N	PY	PY	Y	Y	Y	Y	Y	N	Y	Y	Y	Y	Y	Y	Moderate
Tong et al., 2012	PY	N	N	PY	N/A	N/A	N	N	PY	Y	Y	N	N	Y	N	Y	Critically low
Little et al., 2018	Y	N	PY	Y	N/A	N/A	PY	PY	Y	Y	Y	Y	Y	Y	Y	Y	Low
Angelillo and Villari, 1999	PY	N	PY	PY	Y	Y	Y	Y	Y	N	Y	Y	Y	Y	Y	N	Moderate
Loomis et al., 1999	PY	N	N	N	N/A	N/A	N/A	N/A	N	N	Y	N	N	Y	N	N	Critically low
Wartenberg, 1998	N	N	N	PY	N/A	N/A	N/A	N/A	N	Y	Y	Y	Y	Y	Y	Y	Critically low
Washburn et al., 1994	Y	N	PY	Y	Y	Y	Y	Y	Y	N	Y	Y	Y	Y	Y	N	Moderate
Zhao et al., 2014	Y	N	Y	Y	Y	Y	Y	Y	Y	Y	Y	Y	Y	Y	Y	Y	Moderate
Filippini et al. 2019	Y	N	Y	Y	Y	Y	Y	Y	Y	Y	Y	Y	Y	Y	Y	Y	Moderate
Filippini et al. 2015	Y	N	Y	Y	N/A	N/A	N/A	N/A	Y	Y	Y	Y	Y	Y	Y	N	Low
Chen et al., 2015	Y	N	Y	PY	Y	Y	Y	Y	Y	Y	Y	Y	Y	Y	Y	Y	Moderate
Van Maele-Fabry et al., 2019	Y	N	PY	Y	Y	Y	Y	Y	Y	Y	Y	Y	Y	Y	Y	Y	Moderate
Vinson et al., 2011	PY	N	PY	Y	Y	Y	Y	Y	Y	N	Y	Y	Y	Y	Y	PY	Low

Turner et al., 2010	Y	Y	Y	Y	Y	Y	Y	Y	Y	N	Y	PY	Y	Y	Y	N	Moderate
Wigle et al., 2009	Y	Y	Y	Y	Y	Y	Y	Y	Y	N	Y	PY	Y	Y	Y	N	Moderate
Karalexi et al., 2017	Y	Y	Y	Y	N/A	N/A	N/A	N/A	Y	N	Y	N	N	Y	Y	Y	Low
Latino-Martel et al., 2010	Y	N	Y	PY	Y	Y	Y	Y	N	Y	Y	N	N	Y	Y	Y	Low
Thomopoulos et al., 2015	Y	Y	Y	PY	Y	Y	Y	Y	Y	N	Y	N	N	Y	Y	N	Low
Cheng et al., 2014	Y	N	Y	PY	Y	Y	Y	Y	N	N	Y	Y	Y	Y	Y	Y	Moderate
Klimentopoulou et al., 2012	Y	N	Y	Y	Y	Y	Y	Y	Y	N	Y	Y	Y	Y	Y	Y	Moderate
Chunxia et al., 2019	Y	N	Y	Y	Y	Y	PY	Y	N	Y	Y	N	N	Y	N	Y	Low
Zhou et al., 2014	Y	N	Y	Y	Y	Y	Y	Y	Y	Y	Y	Y	Y	Y	Y	Y	Moderate
Milne et al., 2011	PY	N	PY	PY	N/A	N/A	N/A	N/A	N	Y	Y	N	N	Y	N	Y	Critically low
Liu et al. 2011	PY	N	N	PY	N/A	N/A	N/A	N/A	Y	N	Y	Y	Y	Y	Y	N	Critically low
Cao et al., 2020	Y	N	Y	Y	Y	Y	Y	Y	Y	N	Y	Y	Y	Y	Y	Y	Moderate
Hargreave et al., 2014	Y	N	Y	PY	N/A	N/A	N/A	N/A	PY	Y	Y	Y	Y	Y	Y	Y	Low
Ismail et al., 2019	Y	N	Y	Y	Y	Y	Y	Y	Y	Y	Y	Y	Y	Y	Y	Y	Moderate
Goh et al., 2007	PY	N	Y	Y	N/A	N/A	N/A	N/A	PY	Y	Y	Y	Y	Y	Y	Y	Low
Urayama et al., 2010	Y	N	Y	PY	N/A	N/A	N/A	N/A	Y	Y	Y	Y	Y	Y	Y	Y	Low
Martin et al., 2005	Y	N	Y	Y	N	N	N	N	Y	Y	Y	Y	Y	Y	Y	Y	Low
Amitay et al., 2015	Y	N	Y	Y	Y	Y	Y	Y	Y	N	Y	Y	Y	Y	Y	Y	Moderate
Kwan et al., 2004	PY	N	Y	PY	N/A	N/A	N/A	N/A	Y	N	Y	Y	Y	Y	Y	N	Low
Kinlen, 2012	Y	N	Y	PY	N	N	N	N	PY	N	Y	N	N	Y	PY	N	Critically low
Wang et al., 2018	Y	N	Y	Y	Y	Y	Y	Y	PY	Y	Y	PY	PY	Y	Y	Y	Moderate
Hjalgrim et al., 2003	Y	N	Y	Y	Y	Y	Y	Y	Y	Y	Y	Y	Y	Y	Y	N	Moderate
Caughey et al., 2009	Y	N	Y	PY	N/A	N/A	N/A	N/A	PY	N	Y	PY	PY	Y	Y	N	Critically low

Yan et al., 2020	Y	N	Y	Y	Y	Y	Y	Y	Y	Y	Y	Y	Y	Y	Y	Y	Moderate
Huang et al., 2016	Y	PY	Y	Y	Y	Y	Y	Y	Y	Y	Y	PY	PY	Y	Y	Y	Moderate
Sun et al., 2014	Y	N	Y	Y	Y	Y	Y	Y	Y	N	Y	Y	Y	Y	Y	N	Moderate
Seomun et al., 2021	Y	N	Y	Y	N/A	N/A	Y	Y	Y	Y	Y	N	N	Y	Y	Y	Moderate

Abbreviations: N = no; PECO = population, exposure, comparator, outcome; PY = partial yes; RoB = risk of bias; Y = yes

[1] Did the research questions and inclusion criteria for the review include the components of PECO?

[2] Did the report of the review contain an explicit statement that the review methods were established prior to the conduct of the review and did the report justify any significant deviations from the protocol?

[3] Did the review authors explain their selection of the study designs for inclusion in the review?

[4] Did the review authors use a comprehensive literature search strategy?

[5] Did the review authors perform study selection in duplicate?

[6] Did the review authors perform data extraction in duplicate?

[7] Did the review authors provide a list of excluded studies and justify the exclusions?

[8] Did the review authors describe the included studies in adequate detail?

[9] Did the review authors use a satisfactory technique for assessing the risk of bias (RoB) in individual studies that were included in the review?

[10] Did the review authors report on the sources of funding for the studies included in the review?

[11] If meta-analysis was performed did the review authors use appropriate methods for statistical combination of results?

[12] If meta-analysis was performed, did the review authors assess the potential impact of RoB in individual studies on the results of the meta-analysis or other evidence synthesis?

[13] Did the review authors account for RoB in individual studies when interpreting/ discussing the results of the review?

[14] Did the review authors provide a satisfactory explanation for, and discussion of, any heterogeneity observed in the results of the review?

[15] If they performed quantitative synthesis did the review authors carry out an adequate investigation of publication bias (small study bias) and discuss its likely impact on the results of the review?

[16] Did the review authors report any potential sources of conflict of interest, including any funding they received for conducting the review?

[17] Shea et al. 2017. AMSTAR 2: a critical appraisal tool for systematic reviews that include randomised or non-randomised studies of healthcare interventions, or both. *(5)* For each Yes, 1.0 point was given, and for each Partial Yes, 0.5 points was given. The total sum was then divided by the number of questions answered.
